# Applying three different methods of measuring *CTDI*_*free air*_ to the extended CTDI formalism for wide‐beam scanners (IEC 60601–2–44): A comparative study

**DOI:** 10.1002/acm2.12363

**Published:** 2018-06-14

**Authors:** Robert Bujila, Love Kull, Mats Danielsson, Jonas Andersson

**Affiliations:** ^1^ Medical Radiation Physics and Nuclear Medicine Karolinska University Hospital Stockholm Sweden; ^2^ Department of Physics Royal Institute of Technology Stockholm Sweden; ^3^ Department of Radiation Physics Sunderby Hospital Luleå Sweden; ^4^ Department of Radiation Sciences Umeå University Umeå Sweden

**Keywords:** computed tomography, computed tomography dose index, dosimetry

## Abstract

**Purpose:**

The weighted CT dose index (*CTDI*_*w*_) has been extended for a nominal total collimation width (*nT*) greater than 40 mm and relies on measurements of $CTDI_{free\;air}$. The purpose of this work was to compare three methods of measuring $CTDI_{free\;air}$ and subsequent calculations of *CTDI*_*w*_ to investigate their clinical appropriateness.

**Methods:**

The $CTDI_{free\;air}$, for multiple *nT*s up to 160 mm, was calculated from (1) high‐resolution air kerma profiles from a step‐and‐shoot translation of a liquid ionization chamber (LIC) (considered to be a dosimetric reference), (2) pencil ionization chamber (PIC) measurements at multiple contiguous positions, and (3) air kerma profiles obtained through the continuous translation of a solid‐state detector. The resulting $CTDI_{free\;air}$ was used to calculate the *CTDI*_*w*_, per the extended formalism, and compared.

**Results:**

The LIC indicated that a 40 mm *nT* should not be excluded from the extension of the *CTDI*_*w*_ formalism. The solid‐state detector differed by as much as 8% compared to the LIC. The PIC was the most straightforward method and gave equivalent results to the LIC.

**Conclusions:**

The *CTDI*_*w*_ calculated with the latest CTDI formalism will differ most for 160 mm *nT*s (e.g., whole‐organ perfusion or coronary CT angiography) compared to the previous CTDI formalism. Inaccuracies in the measurement of $CTDI_{free\;air}$ will subsequently manifest themselves as erroneous calculations of the *CTDI*_*w*_, for *nT*s greater than 40 mm, with the latest CTDI formalism. The PIC was found to be the most clinically feasible method and was validated against the LIC.

## 1 INTRODUCTION

Computed tomography (CT) is associated with relatively high radiation doses. The accurate estimation of radiation exposure is therefore a major priority within the medical physics community. The CT dose index (CTDI) is a ubiquitous dose quantity used in CT. The CTDI represents an approximation of the average absorbed dose in a standard geometry (PMMA cylinder with a diameter of 32 cm for body scans or 16 cm for head scans and a length of approximately 14 cm) in a central rotation, including scatter contributions from adjacent rotations.^1^ Since the CTDI represents the average absorbed dose in a standard geometry, it should not be confused with patient dose; however, it does provide useful and comparative information about the output of CT scanners.^2^

In practice, the CTDI is determined by integrating the dose profile from a single axial scan along the z‐direction, $D\left(z\right)$, and normalizing that integral with the nominal total collimation width (*nT*) of the scan. When the CTDI was first proposed in 1981^3^ and subsequently became a standard dose quantity in CT,^4^ it was common with relatively narrow beam CT scanners. In 1999, the International Electrotechnical Commission (IEC) published the European Standard “Particular requirements for the safety of X‐ray equipment for computed tomography” where the integration length of the CTDI was fixed to z=±50mm (*CTDI*_100_) and it also stated that $D\left(z\right)$ should be measured in air kerma (*K*_*air*_).^5^ A 100‐mm pencil ionization chamber is appropriate to use according to this definition of the CTDI. In that same European Standard, equations for calculating the weighted CTDI (*CTDI*_*w*,100_) from central and peripheral *CTDI*_100_ measurements in the reference phantoms is given. The *CTDI*_*w*,100_ represents the average *CTDI*_100_ across a reference phantom in the axial plane.^4^

Nowadays, there are CT scanners with wide‐beam geometries that facilitate axial scans with a detector coverage up to 160 mm in a single a rotation. This technique allows for entire organs to be obtained in a single volume with short acquisition times (commensurate with the CT scanner's rotation time). This type of scanning is useful when imaging dynamic processes including, among other types of studies, brain perfusion, as well as coronary CT angiograms.^6^, ^7^ Take note that perfusion scans often require many passes and the accumulated radiation doses can be high.

Boone calculated the CTDI efficiency $\left(\varepsilon_{\mathrm{CTDI}}=\;\frac{CTDI_{100}}{CTDI_{\infty }}\right)$ for a number of *nT*s in both the reference head and body phantoms and found that the *CTDI*_100_ underestimated *CTDI*_∞_ appreciably, even for narrow *nT*s, due to the truncation of $D\left(z\right)$; the underestimation becomes more apparent as *nT* increases.^8^ For that reason, applying the first definition of the CTDI to scans with wide nominal total collimation widths would severely underestimate the radiation output of a wide‐beam acquisition.

In order to provide more accurate determinations of the output of CT scanners with wide *nT*s, Geleijns et al. 9 proposed extending the integration length of CTDI measurements by using a 300‐mm pencil ionization chamber and 350‐mm wide reference phantoms (*CTDI*_300_) for scanners with wide *nT*s; however, this method never saw widespread adoption in clinic practice. In its third edition of the aforementioned European Standard, the IEC modified the definition of the *CTDI*_100_ (to accommodate wide‐beam scanners) as the integral of $D\left(z\right)$ (100 mm integration length) normalized by *nT* or 100 mm, whichever is less.^10^ This definition of the *CTDI*_100_ has been shown to approximate the *CTDI*_300_ within ±10% for both the reference head and body phantoms for a wide range of tube voltages and shaped filters with an *nT* of 160 mm.^9^ In the third edition of the European Standard, the IEC also defined the $CTDI_{free\;air}$ as the *CTDI*_100_ measured in the center of the axial plane without a phantom.^10^

In 2012, the IEC further modified the definition of the *CTDI*_100_ for nominal total collimation widths that exceed 40 mm, in a first amendment to the third edition of its European Standard.^11^ It should be noted that for nominal total collimation widths that are less than or equal to 40 mm, the definition of the *CTDI*_100_ remains the same as previous editions of the European Standard. The redefinition of the *CTDI*_100_ for wide beams utilizes the assumption that the ratio of $CTDI_{free\;air}$ (with appropriate integration lengths to capture $D\left(z\right)$) between two nominal total collimation widths is equal to the ratio of *CTDI*_100_ with the same *nT*s.^12^ Using this relationship, the *CTDI*_100,*nT*>40 *mm*_ is approximated by multiplying a measured *CTDI*_100,*nT*≤40 *mm*_ with the ratio of $CTDI_{free\;air,nT>40\;mm}$ and $CTDI_{free\;air,nT\leq 40\;mm}$.

To the best of our knowledge, no one has compared different methods of measuring $CTDI_{free\;air}$ and their subsequent calculation of the *CTDI*_*w*_ per the extended formalism. The purpose of this work was to use three different methods of measuring $CTDI_{free\;air}$ and apply those measurements to the latest amendment of the IEC Standard,^11^ for a range of *nT*s up to 160 mm. The $CTDI_{free\;air}$ was first determined using an advanced method, high‐resolution step‐and‐shoot translation of a liquid ionization chamber, which is considered to be a dosimetric reference. The advanced method was compared to more clinically feasible methods of determining the $CTDI_{free\;air}$, namely, (1) using multiple contiguous positions to extend the integration length of a 100‐mm pencil ionization chamber and (2) continuous translation of a real‐time solid‐state detector.

## 2 METHODS AND MATERIALS

### 2.A CTDI formalism

When first proposed, the CTDI was defined as,

(1) $$CTDI_{\infty }=\frac{1}{\mathrm{nT}}\int_{-\infty }^{\infty }D\left(z\right)dz,$$

where $D\left(z\right)$ is a dose profile along the longitudinal axis, *z*, centered at *z* = 0. The number of detector channels and the width of each channel are *n* and *T*, respectively.^3^ Note that *nT* represents the nominal total collimation width of the scan and in the early days of CT it was common with single slice scanners (*n* = 1). In the first edition of IEC 60601–2–44,^5^ the CTDI was defined for an integration length of 100 mm as,

(2) $$CTDI_{100}=\frac{1}{\mathrm{nT}}\int_{-50\;mm}^{50\;mm}D\left(z\right)dz.$$

In that same edition of the IEC standard, the weighted CTDI was defined as,

(3) $$CTDI_{100,w}=\;\frac{1}{3}CTDI_{100,c}+\;\frac{2}{3}CTDI_{100,p},$$

where *c* and *p* denote measurements of the *CTDI*_100_ in the central and peripheral holes of the CTDI reference phantoms, respectively. In practice, the *CTDI*_100,*p*_ is the mean *CTDI*_100_ from the four peripheral holes in the reference CTDI phantoms. In the first amendment to the third edition of IEC 60601–2–44,^11^ the *CTDI*_100_ was redefined for *nT*s that exceed 40 mm as,

(4) $$CTDI_{100,nT>40\;mm}=CTDI_{100,ref}x\left(\frac{CTDI_{free\;air,\;nT}}{CTDI_{free\;air,\;ref}}\right),$$

where the subscript *ref* represents a reference nT that is equal to or less than 40 mm. In that same European Standard,^11^ the CTDI measured in free air was defined as,

(5) $$CTDI_{free\;air}=\;\frac{1}{\mathrm{nT}}\int_{-L/2}^{L/2}D\left(z\right)dz,$$

where *L* is at least the *nT* of a single scan plus an additional 40 mm divided on both sides (nT+40mm). Furthermore, it is stated that the $CTDI_{free\;air}$ should not be calculated with an integration length below 100 mm.^11^

Contemporary CT scanners are obligated to present the volume CTDI (*CTDI*_*vol*_) in accordance with the first amendment to the second edition of IEC 60601–2–44.^13^ The *CTDI*_*vol*_ accounts for table translations that produce overlapping or gapped exposure(s). The *CTDI*_*vol*_ is calculated as $\frac{CTDI_{100,w}}{\Delta }$, where Δ is the longitudinal table translation between scans divided by the nominal beam width for axial modes or the CT pitch factor for spiral modes. However, in the continuation of this work, only the *CTDI*_100,*w*_ will be considered, which is equivalent to the *CTDI*_*vol*_ where Δ = 1 and an axial scan mode has been used.

To leverage a better understanding of how the first amendment to the third edition of IEC 60601–2–44 can be implemented in practice to measure the *CTDI*_100,*w*_ for *nT*s greater than 40 mm, the following steps can be followed:

1. Measure the *CTDI*_100,*c*_ and *CTDI*_100,*p*_ for a reference (*ref*) *nT* equal to or less than 40 mm, resulting in *CTDI*_100,*ref*,*c*_ and *CTDI*_100,*ref*,*p*_.
2. Measure the $CTDI_{free\;air}$ [Eq. (5)] with the same reference (*ref*) *nT* as step 1, resulting in $CTDI_{free\;air,ref}$.
3. Measure the $CTDI_{free\;air}$ [Eq. (5)] with an *nT* that is greater than 40 mm, resulting in $CTDI_{free\;air,nT}$.
4. Calculate the ratio of $\frac{CTDI_{free\;air,nT}}{CTDI_{free\;air,ref}}$ and multiply with *CTDI*_100,*ref*,*c*_ and *CTDI*_100,*ref*,*p*_ respectively. This will result in $CTDI_{100,nT,\;c}$ and $CTDI_{100,nT,\;p}$.
5. Use Eq. (3) and the results from step 4 to calculate *CTDI*_100,*nT*,*w*_.

### 2.B CT scanner

All measurements were made on a Revolution CT (GE Healthcare, Waukesha, WI, USA). In addition to this scanner being able to perform volume scans with *nT*s up to 160 mm, the CTDI is reported with the formalism from the first amendment to the 3rd edition of IEC 60601–2–44 in the scanner's latest software releases (since version 15MW43.x). The technique parameters used in this study, Table 1, reflect parameters that are given in the acceptance testing section of the scanner's Technical Reference Manual (TRM).^14^ All measurements were made in clinical mode. Note that the reference *nT* in Eq. (4) is 5 mm on this scanner.

**Table 1:** Technique parameters used in this study

Scan mode	Tube Voltage [kVp]	Tube current [mA]	Focal spot size	Rotation time [s]	Shaped filter	Nominal total collimation width [mm]
Axial	120	400	Large	1	Large	5, 40, 80, 120, 160

### 2.C Determination of *CTDI*_100,*nT*,*w*_

In this work, the *CTDI*_100,*nT*>40 *mm*_, Eq. (4), is calculated using $CTDI_{free\;air,nT}$ that is measured with three different measurement systems. The three different measurement systems used to obtain $CTDI_{free\;air,nT}$ are subsequently used to determine *CTDI*_100,*nT*,*w*_.

#### 2.C.1 *CTDI*_free air,*nT*_ determined with high‐resolution air kerma profiles measured with a liquid ionization chamber and a step‐and‐shoot methodology

**Figure 1:**
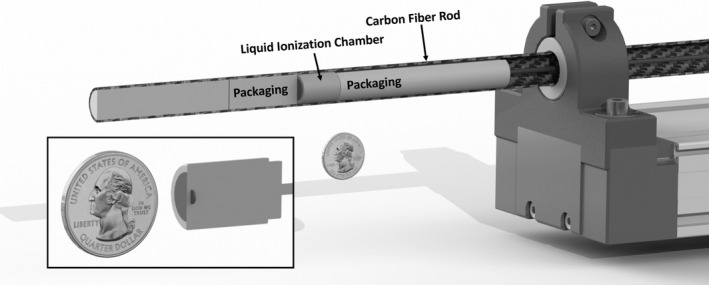
A depiction of the rig that was developed to step a liquid ionization chamber so that high‐resolution air kerma profiles could be measured to calculate $CTDI_{free\;air}$. In the lower left, the liquid ionization chamber has been cut out where the sensitive volume of the detector is represented by the imbedded dark disk. A US quarter dollar coin has been used as a size reference.

A measurement rig, designed, and assembled by LoniTech AB (Luleå, Sweden), was used to acquire air kerma profiles with a step‐and‐shoot methodology, see Fig. 1. A microLion (PTW GmbH, Freiburg, Germany) liquid ionization chamber (LIC) with a sensitive volume of 0.002 cm^3^ (cylindrical diameter of 2.5 mm and cylindrical height of 0.35 mm) was packaged into a minimally attenuating carbon fiber rod. The packaged LIC received an RQT‐9 calibration (full field irradiation) from the National Metrology Laboratory (national secondary standards laboratory for the dosimetric quantity air kerma) at the Swedish Radiation Safety Authority. A Unidos Universal Dosemeter (PTW GmbH, Freiburg, Germany) was used for the LIC charge measurement readings. All LIC measurements were corrected for temperature 15 with values that were obtained with a temperature sensor during the exposure at each step of the translation. The temperature sensor was positioned in proximity to the end of the patient table where the LIC was stepped through the gantry.

The packaged LIC was coupled to a linear actuator that provided a stepwise translation in the longitudinal direction with a positioning accuracy better than 0.2 mm. Prior to the air kerma measurements, the carbon fiber rod was extended fully (500 mm) and a 160‐mm volume scan (256 images) was taken to ensure that the stepwise translation was centered (*x* = 0, *y* = 0) and was free from rotational pitch and yaw (rotation about the x‐ and y‐axes of the scanner, respectively). Depending on the *nT*, different profile lengths and step intervals were used (see Table 2). The profile measurements did not have equal step intervals, the interval was tighter at locations where the air kerma varied greatly, at, for example, step locations in the penumbra of the radiation field where the air kerma rapidly increased or decreased. The $CTDI_{free\;air,nT}$, Eq. (5), was calculated for each *nT* using the *trapz* function (numerical integration using the trapezoidal method) in MATLAB 2016b (The Mathworks Inc., Nattick, MA, USA) to integrate the air kerma profile, $D\left(z\right)$.

**Table 2:** Profile length and number of steps used when measuring air kerma profiles using a step‐and‐shoot methodology with the liquid ionization chamber

Nominal total collimation width [mm]	Integration length [mm]	Steps
5	100 (−50 mm to 50 mm)	93
40	300 (−150 mm to 150 mm)	124
80	300 (−150 mm to 150 mm)	130
120	400 (−200 mm to 200 mm)	153
160	490 (−245 mm to 245 mm)	153

The type A uncertainty associated with this method was estimated by measuring 5 air kerma profiles using the technique parameters in Table 1 and a 5‐mm *nT*. The number of measurement points was reduced to 40 and covered a length of 100 mm (−50 mm to 50 mm). The step interval was adapted to parts of the profile that increased or decreased rapidly. It would not have been feasible to estimate the type A uncertainty for all *nT*s.

#### 2.C.2 *CTDI*_*free air,nT*_ determined with pencil ionization chamber measurements at multiple contiguous locations

In IAEA Human Health Reports No. 5,^12^ a method is provided to measure the $CTDI_{free\;air,nT}$ for *nT*s exceeding 40 mm. In our practical implementation of this method, a 100‐mm pencil ionization chamber (PIC) is suspended in the longitudinal direction (*x* = 0, *y* = 0) using a minimally attenuating carbon fiber rod. Depending on the *nT*, the PIC is stepped into different contiguous locations between exposures to envelope the entire radiation field. The positions of the contiguous locations that are recommended by the IAEA to extend the integration length of the $CTDI_{free\;air,nT>40\;mm}$ using a PIC are presented in Table 3. These recommended positions from IAEA are consistent with recommendations by Platten et al.^16^ Furthermore, GE states alternative PIC positions in the scanner's TRM^14^ to extended the integration length of $CTDI_{free\;air,nT>40\;mm}$, these positions are also provided in Table 3. The GE positions differ greatly from the IAEA positions; however, the GE positions are based on recommendations from the IEC (personal communication with GE, December 2016). The CT scanner's table translation was used to move the PIC into contiguous positions.

**Table 3:** Contiguous positions of the 100‐mm pencil ionization chamber that can be used to calculate $CTDI_{free\;air,nT}$ for nominal total collimation widths (*nT*) that exceed 40 mm. The positions assume that the central location of the pencil ionization chamber is located at z = 0 on the scanner

Nominal total collimation width [mm]	Integration length [mm]	IAEA positions 12 [mm]	GE positions 14 [mm]
5	100	0	0
40	100	0	0
80	200	−50, 50	−10, 90
120	200	−50, 50	−30, 70
160	300	−100, 0, 100	−50, 50, 150

**Figure 2:**
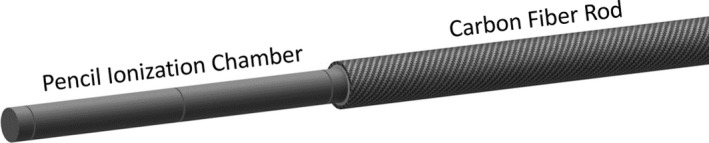
This figure depicts how the pencil ionization chamber was attached to a carbon fiber rod so that measurements of $\mathrm{CTD}I_{free\;air}$ could be made.

A carbon fiber rod was attached to the cable end of a 100 mm RC3CT PIC (Radcal Corporation, Monrovia, CA, USA), see Fig. 2. This pencil ionization chamber also received an RQT‐9 calibration from the National Metrology Laboratory at the Swedish Radiation Safety Authority. The same electrometer that was used for the LIC measurements was used for the PIC measurements. The ambient temperature and pressure were measured during each exposure so that a standard air pressure and temperature correction to the measurements with the PIC could be made. The central marking of the PIC was positioned in isocenter (*x* = 0, *y* = 0, *z* = 0) and a 160‐mm volume scan (256 images) was used to ensure that the PIC was properly centered and free from rotational pitch and yaw. At each PIC location and *nT*, five exposures were made. The mean value of the five exposures at each location was used in the continuation of this work. Additionally, the type A uncertainty of this method was analyzed.

#### 2.C.3 *CTDI*_*free air,nT*_ determined with solid‐state detector system measurements during continuous longitudinal translation

All instruments and software used for the measurement of $CTDI_{free\;air,nT}$ using a real‐time solid‐state detector and continuous translation are manufactured by RTI Electronics (Mölndal, Sweden). A CT Dose Profiler (CTDP) is a real‐time solid‐state detector designed for CT dosimetry applications. The active length of the detector's sensor along the longitudinal axis is 250 μm and it is packaged in an aluminum rod. The CTDP was connected to a Black Piranha electrometer. The CTDP received an RQT‐9 calibration at RTI Electronics’ calibration laboratory.

A device called the Mover (manufactured by RTI Electronics) was used to translate the CTDP continuously along the longitudinal axis of the CT scanner. The Mover has a motor that drives a wire that is connected to the end of the CTDP. The CTDP is translated through a plastic tube that is placed along the longitudinal axis of the scanner. All measurements were made in the pull direction (toward the Mover's motor) with a translation speed of 83.3 mm/s. The Mover's translation speed was calibrated according to an application note (AN‐034, June 2015) provided by RTI Electronics.

The Mover was centered in the CT scanner using the positioning lasers and a wide volume scan was used to ensure that the setup was centered and free from rotational pitch and yaw. Five air kerma profile measurements were made for all *nT*s presented in Table 1. The mean value of the five measurements was used in the continuation of this work and the type A uncertainty was calculated. The electrometer (Black Piranha) and the Mover were controlled using the accompanying software package Ocean 2014 version 2016.01.18.199. The measured air kerma profiles were exported from Ocean 2014 and MATLAB 2016b was used to determine the $CTDI_{free\;air,nT}$ according to Eq. (5) in a similar manner to the measurements made with the LIC.

#### 2.C.4 Calculations of the weighted CTDI (*CTDI*_100,*nT*,*w*_)

A standard body CTDI phantom (model 007, CIRS Inc., Norfolk, VA, USA) with a diameter of 32 cm and a length of 14 cm was used to measure the *CTDI*_100,*ref*,*w*_, Eq. (3), with a reference *nT* of 5 mm. The CTDI phantom was placed approximately 1 m toward the middle of the patient table from the end that is closest to the gantry. The CTDI phantom was imaged with a volume scan to ensure that it was centered properly and was free from rotational pitch and yaw. The $CTDI_{100,5\;mm}$ was measured five times in each of the central and peripheral holes using the same measurement system in section 2.3.2. A temperature and pressure correction was made for each measurement. The mean value of each set of five measurements was used in the determination of the $CTDI_{100,\;\;5\;mm,w}$, Eq. (3). The $CTDI_{100,5\;mm,\;p}$ is the average value of all measurements in the peripheral holes of the CTDI phantom.

Using the same measurement method that was employed for the $CTDI_{100,\;5\;mm,w}$, the $CTDI_{100,\;nT,w}$ was measured for the remainder of the collimations according to the third edition of IEC 60601–2–44,^10^ where *nT* in Eq. (2) is equal to the *nT* or 100 mm, whichever is less. Measurements of the $CTDI_{100,\;nT,w}$ are compared using both the third edition and the first amendment to the third edition of IEC 60601–2–44.^11^

## 3 RESULTS

**Figure 3:**
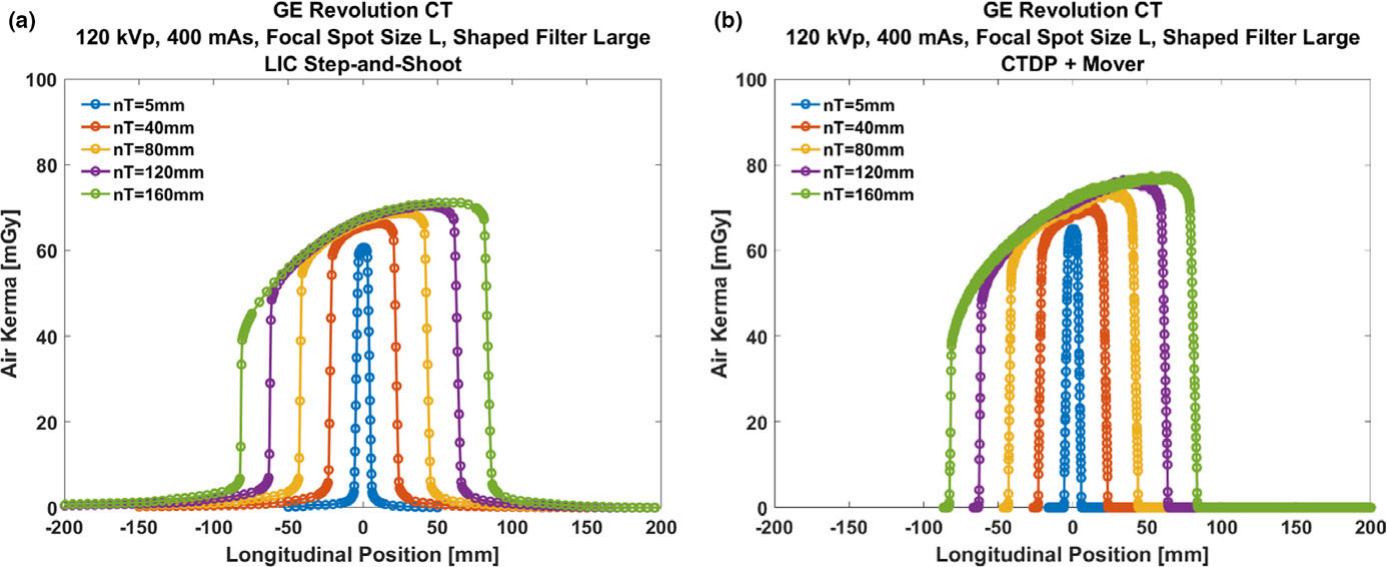
Air kerma profiles along the z‐direction for different nominal total collimation widths (*nT*) using (a) LIC and a step‐and‐shoot methodology and (b) CTDP and a continuous translation with the RTI Mover.

The air kerma profiles that were obtained using a step‐and‐shoot methodology with the LIC is presented in Fig. 3(a). Figure 3(b) presents air kerma profiles that were obtained using the continuous translation of the CTDP (solid‐state detector). Note that the air kerma profiles in Fig. 3(b) are free from scatter tails. The scatter tails are removed in the Ocean 2014 software package since they are considered to have been caused by the aluminum rod in which the solid‐state detector is packaged in (personal communication with RTI Electronics, December 2016).

The $\frac{CTDI_{free\;air,nT}}{CTDI_{free\;air,ref}}$ ratios for different *nT*s that were determined using the three different measurement methods considered in this work are presented in Table 4. Included in the table are expectation values quoted from the CT scanner's TRM.^14^ The absolute value of the $CTDI_{free\;air,\;nT}$ (in mGy) measured with each method is provided next to each ratio in parentheses. The CTDP measurement method resulted in absolute $CTDI_{free\;air,nT}$ values that were greater than the other measurement methods as well as the TRM. For all *nT*s the absolute value of the $CTDI_{free\;air,nT}$ (given in parentheses) for each *nT* are within 8% of each other irrespective of measurement method. The $\frac{CTDI_{free\;air,nT}}{CTDI_{free\;air,ref}}$ ratios differed at most by 4.5% of each other irrespective omeasurement method for *nT*s greater than 40 mm.

**Table 4:** The ratio $\frac{CTDI_{free\;air,nT}}{CTDI_{free\;air,ref}}$ for the different measurement methods studied in this work where the reference nominal total collimation width (nT)is 5 mm. The absolute value of the $CTDI_{free\;air}$ is given in parentheses with the unit mGy

Nominal total collimation width [mm]	GE TRM 14	PIC GE positions section 2.3.2	PIC IAEA positions section 2.3.2	LIC section 2.3.1	CTDP section 2.3.3
5	1.00 (123.9)	1.00 (122.1)	1.00 (122.1)	1.00 (120.5)	1.00 (125.0)
40	0.61 (75.8)	0.62 (75.3)	0.62 (75.3)	0.64 (76.5)	0.63 (78.6)
80	0.61 (75.0)	0.60 (73.4)	0.60 (73.5)	0.61 (73.0)	0.62 (77.5)
120	0.59 (72.5)	0.58 (71.0)	0.58 (70.9)	0.59 (70.6)	0.61 (76.0)
160	0.57 (70.8)	0.57 (69.3)	0.57 (69.4)	0.57 (68.1)	0.59 (74.1)

Table 5 presents calculations of the $CTDI_{100,\;nT,w}$ according to the third edition of IEC 60601–2–44.^10^ Alongside those calculations are expectation values of the $CTDI_{100,\;nT,w}$ that are provided in the scanner's TRM. The values presented in Table 5 represent calculations of $CTDI_{100,\;nT,w}$ according to Eq. (2), where *nT* is set to 100 mm for *nT*s that are greater than 100 mm. The measured values of $CTDI_{100,\;nT,w}$ are consistently lower than the expectation values stated in the scanner's documentation by 4–8%, depending on the *nT*.

**Table 5:** Values of $CTDI_{100,\;nT,w}$ (32 cm) that were calculated through measurements according to the third edition of IEC 60601–2–44. This table also shows expectation values of $CTDI_{100,\;nT,w}$ that were obtained from an older revision of the scanner's technical reference manual.^17^

Nominal total collimation width [mm]	GE TRM 17 [mGy]	Measured $CTDI_{100,\;nT,w}$ [mGy]
5	45.3	42.0
40	27.3	26.2
80	25.2	24.3
120	27.3	25.9
160	30.6	28.3

The $CTDI_{100,\;nT,w}$ was calculated with Eq. (4) using the $\frac{CTDI_{free\;air,nT}}{CTDI_{free\;air,ref}}$ ratios (Table 4) measured with the three different methods in this study. These results of the $CTDI_{100,\;nT,w}$ are presented in Table 6. Equation (4) is intended for *nT*s greater than 40 mm. For that reason, $CTDI_{100,\;nT,w}$ for *nT*s of 5 and 40 mm are not presented in Table 5. Take note that the $CTDI_{100,\;nT,w}$, for an *nT* of 5 mm, (Table 5) is used as the $CTDI_{100,\;ref,w}$ for the calculations presented in Table 6. Alongside the $CTDI_{100,\;nT,w}$ in Table 6, expectation values from the scanner's TRM have also been included. There is a slight difference between the expectation values for $CTDI_{100,\;nT,w}\;$between the different revisions of the scanner's TRM for *nT*s of 5 and 40 mm. In the scanner's latest revision of the TRM,^14^ the expectation values for $CTDI_{100,\;nT,w}$ are 44.5 and 27.2 mGy for the *nT*s of 5 and 40 mm, respectively.

**Table 6:** The $CTDI_{100,\;nT,w}$ (32 cm) values that were calculated according to the first amendment to the third edition of IEC 60601–2–44 11 using different methods of measuring $CTDI_{free\;air}.$

Collimation [mm]	GE TRM 14	PIC GE positions section 2.3.2	PIC IAEA positions section 2.3.2	LIC section 2.3.1	CTDP section 2.3.3
80	27.0	25.2	25.3	25.4	26.0
120	26.2	24.4	24.4	24.6	25.5
160	25.4	23.8	23.8	23.7	24.9

The greatest difference between any of the PIC, LIC, and CTDP measurements in Table 6 to the scanner's TRM is −7.5% for the PIC (IAEA Positions), with an *nT* of 120 mm. The greatest difference between any of the PIC results, both GE and IAEA positions, compared to the LIC results in Table 6 is 0.8%. However, the greatest difference between any of the CTDP results compared to the LIC results in Table 6 is 4.5% with an *nT* of 160 mm.

The relative standard uncertainty of $CTDI_{free\;air,\;nT}$ for the PIC and CTDP measurements steadily decreased as the *nT* increased. Take note, the relative standard uncertainty of $CTDI_{free\;air,\;nT}$ that required multiple PIC positions was calculated using a standard error propagation. The PIC measurement method, both the GE and IAEA positions, had a relative standard uncertainty between 0.04 and 0.01% for all *nT*s. The CTDP method had a relative standard uncertainty between 0.6 and 0.1% for all *nT*s. The relative standard uncertainty for the LIC method was estimated to be 0.2% for an *nT* of 5 mm. For *nT*s greater than 5 mm, the uncertainty is expected to be lower, since there are more measurement points along the air kerma profiles (better statistics).

## 4 DISCUSSION

In this work, different methods of measuring $CTDI_{free\;air,nT}$ have been investigated and subsequently used to calculate the $CTDI_{w,\;nT}$ for wide‐beam CT dosimetry according to the CTDI formalism in the first amendment to 3rd edition of IEC 60601–2–44.^11^ As the amended wide‐beam CT dosimetry formalism has recently been implemented on CT scanners, it necessitates that clinical Medical Physicists have a good understanding of how different methods of measuring $CTDI_{free\;air}$ may impact the accuracy of their results.

According to the investigated scanner's TRM, the maximum allowed deviation for $CTDI_{free\;air}$ measurements is 40% from the stated expected values, which includes uncertainty contributions from variations in tube output, x‐ray beam collimation, dosimetry methodology, and calibration errors.^14^ All methods that were investigated to measure the $CTDI_{free\;air}$ for different *nT*s are well within the 40% tolerance stated by the vendor. However, in the scope of the work, the LIC was considered to be a dosimetric reference and not the CT scanner's TRM. While the PIC methods yielded comparable results of the $CTDI_{free\;air}\;$to the LIC (within 2%), the CTDP method consistently yielded results that were 6%–8% higher than the LIC for *nT*s greater than 40 mm.

Table 5 shows that for *nT*s that are less than 100 mm, the $CTDI_{100,\;nT,w}$ decreases as *nT* increases, which is consistent with the third edition of IEC 60601‐2‐44.^10^ When the *nT* is greater than 100 mm, the $CTDI_{100,\;nT,w}$ increases as the *nT* increases. This can be attributed to the CTDI phantom only having a length of 14 cm. As the *nT* increases, more and more scatter will contribute to the air kerma that the pencil ionization chamber measures. However, the normalization factor (*nT*) in Eq. (2) will remain constant (100 mm) for *nT*s greater than 100 mm. With the first amendment to the third edition of IEC 60601‐2‐44,^11^ the $CTDI_{100,\;nT,w}$ continues to decrease as the *nT* increases above 100 mm. Comparing the third edition with the first amendment to the third edition of IEC 60601‐2‐44 shows that there will be a slight increase in $CTDI_{100,\;nT,w}$ for an *nT* of 80 mm (+5%) and a decrease in $CTDI_{100,\;nT,w}$ for an *nT* of 160 mm (−19%) when using the latest definition of the $CTDI_{100,\;nT,w}$ with the LIC and PIC methods. However, the difference is +7% (80 mm) and −14% (160 mm) for the CTDP method. The values from the TRM for the different versions of the CTDI formalism yields +7% (80 mm) and −20% (160 mm). A difference of −20% between the measured $CTDI_{100,\;nT,w}$ and the $CTDI_{100,\;nT,w}$ displayed on the scanner's console is considered to be a trigger to take remedial action by the Nordic Association of Clinical Physicists (NACP).^17^

It is important to reflect upon that even though the $CTDI_{100,\;nT,w}$ may decrease by 19% using the latest CTDI formalism and an *nT* of 160 mm, the exposure to the patient (and image quality of the examination) will remain the same using the previous CTDI formalism. This should be considered when optimizing, for example, whole‐organ perfusion protocols where the accumulated radiation doses can be high. In the context of patient dose, the CTDI metric has disadvantages in specificity with regard to characterizing the x‐ray beam and the individual patient undergoing an examination. However, the evolution of applied CT dosimetry is built upon the CTDI, most notably in the form of size‐specific dose estimates (SSDE).^18^, ^19^ It is therefore important to continue discussing the theoretical and practical aspects of the CTDI. Further refinements of CT dosimetry, beyond the SSDE, where the x‐ray beam characteristics and contributions from scattered radiation are completely taken into account will require new reference geometries and more detailed descriptions of exposure.^20^

Each of the measurement methods was associated with a relatively low type A uncertainty (below 1% for the CTDP method, below 0.2% for the LIC method and below 0.05% for the PIC method). However, this uncertainty evaluation does not factor in uncertainties in, for example, air kerma calibration factors. In the scope of this work, the step‐and‐shoot method of acquiring air kerma profiles with the LIC is considered to be the most accurate method that was used to obtain $CTDI_{free\;air,nT}$ for the range of *nT*s, both dosimetrically and spatially. The LIC has appreciable energy dependence when compared to other instruments, such as pencil ionization chambers, over a range of radiation qualities. However, the LIC used a calibration (RQT‐9), which closely matches the radiation quality that was used during the measurements. The HVL associated with the RQT‐9 radiation quality is 8.7 mm Al and the HVL along the central ray of the Revolution CT for 120 kVp with the Large shaped filter is 7.6 mm Al.^14^ The downside to this method is that it is very labor intensive to produce the rig and measurements with this method are time consuming (as it requires many exposures to capture the air kerma profiles). For those reasons, it is not likely that this method could be employed in clinical routine. However, the LIC provided a good reference to which the other measurement methods could be compared.

The PIC measurement method(s) focus on extending the use of readily available instruments (100‐mm pencil ionization chambers) so that they may be employed to measure $CTDI_{free\;air,nT}$ for wide *nT*s, in clinical routine. This was done by suspending a pencil ionization chamber free‐in‐air and moving the pencil ionization chamber into up to three contiguous positions. The measurements in each position are summed, and in effect extends the integration length to either 200 or 300 mm, depending on the number of positions used. This method is straightforward and easily practiced in clinical routine and for the most part gave results that were equivalent to the LIC (within the measurement uncertainty). It does however assume that the effective measurement length of the PIC is 100 mm. There could be a systematic uncertainty induced into the measurements if the effective length of the PIC diverges from 100 mm (overlap or gaps between measurements); however, it is outside of the scope of this work to investigate. There was no appreciable difference in the $CTDI_{free\;air,nT}$ between the different (GE's recommended and IAEA's recommended) positions for the different contiguous locations of the PIC.

It was our intention to measure with the LIC, PIC, and CTDP during the same measurement session. However, after reviewing the results from the CTDP, it was concluded that the RTI Mover (continuous translation) device was not properly calibrated and inconsistent results between measurements were obtained. It was not until we could verify that the Mover was properly calibrated from RTI Electronics that another set of measurements were made. Between the different measurement times, the absolute dose of the $CTDI_{free\;air}$ differed appreciably (e.g., between 113 and 125 mGy for an *nT* of 5 mm). The reason for this difference between measurement times is uncertain. It is unlikely that this difference can be attributed to scanner output since similar measurements using the LIC were taken around the same time as the CTDP measurements and showed that the output had changed by no more than 1%. For wide (>40 mm) *nT*s, the CINE function (multiple continuous stationary rotations) was used to provide an exposure that was long enough in time so that the CTDP could be translated through the entirety of the radiation field. The fastest translation speed on the Mover is 83.3 mm/s and would require more than a 2 s exposure (2 rotations) to be able to measure the $CTDI_{free\;air}$ for an *nT* of 160 mm. We do not expect the CINE mode to have impacted the results compared to a axial mode. Furthermore, it was difficult to obtain values of $CTDI_{free\;air,nT}$ directly from the Ocean 2014 software. For that reason, air kerma profiles were exported from Ocean 2014 and calculations of $CTDI_{free\;air,nT}$ were made with MATLAB.

It should be noted that this study is limited by the fact that there are other instruments that could potentially be used to determine the CTDI according to its latest formalism for CT scanners with wide nominal total collimation widths. This includes instruments such as a 300‐mm pencil ionization chamber or stepping a thimble chamber through the beam to acquire air kerma profiles. Furthermore, alternative methods of determining the weighted CTDI, such as using radiochromic film, has also not been investigated.

The measured value of $CTDI_{free\;air,\;nT}$ (and the ratio $\frac{CTDI_{free\;air,nT}}{CTDI_{free\;air,ref}}$) for the 40 mm collimation using the LIC and CTDP methods is greater than the PIC method (see Table 4), as well as the expectation value in the scanner's technical documentation.^14^ Note that the integration length of $CTDI_{free\;air,nT}$ for the LIC method was 300 mm compared to 100 mm (one position) using the PIC. Calculating $CTDI_{free\;air,nT}$ with the LIC, but using a 100 mm integration length (−50 to 50 mm) yields a value of 74.44 mGy or alternatively a $\frac{CTDI_{free\;air,nT}}{CTDI_{free\;air,ref}}$ ratio of 0.62 (the same ratio that was measured using the PIC). Extending the integration length of $CTDI_{free\;air}$ for the 40 mm collimation to 200 mm using 2 PIC positions (−50 and 50 mm) yields a value of 76.94 mGy or a $\frac{CTDI_{free\;air,nT}}{CTDI_{free\;air,ref}}$ ratio of 0.63 which is closer to the value that was obtained using the full integration length (300 mm) of the air kerma profile for the LIC measurement. This could possibly indicate that an integration length of 100 mm for $CTDI_{free\;air}$ measurements for a 40 mm collimation might not include the entire air kerma profile along the longitudinal direction. The additional contribution to the $CTDI_{free\;air,nT}$ that was observed when using an air kerma profile integration length greater than 100 mm, for the 40 mm *nT,* can be caused by several factors including the geometric efficiency (actual beam width is wider than the nominal total collimation width), extra focal radiation, as well as penetration of radiation through the collimator edges.

## 5 CONCLUSIONS

As CT is associated with relatively high radiation doses, it is therefore important for medical physicists to be able to accurately estimate the output from CT scanners. If the radiation output from a scanner is erroneously measured or reported, this could inadvertently lead to unnecessary radiation exposure or degraded image quality, both having unintended consequences. An amendment to the third edition of IEC 60601‐2‐44 has been made to further extend the concept of the *CTDI*_*w*_ to CT scanners with nominal total collimation widths (*nT*) greater than 40 mm. This amendment has recently been implemented on certain CT scanners and clinical Medical Physicists need to update their measurement methodologies accordingly. The amendment relies on measurements of $CTDI_{free\;air}$ with integration lengths that exceed the length of a standard 100‐mm pencil ionization chamber. In this work, three methods of acquiring air kerma profiles to calculate the $CTDI_{free\;air}$, for a range of *nT*s, were implemented and subsequent calculations of the *CTDI*_*w*_ were compared. The measurement methods consisted of:

1. high‐resolution air kerma profiles using a step‐and‐shoot translation of a liquid ionization chamber (considered to be a dosimetric reference),
2. the sum of multiple 100‐mm pencil ionization chamber measurements where the chamber is placed at different contiguous locations in the z‐direction,
3. continuous translation of a real‐time solid‐state detector.

The liquid ionization chamber results suggested that the latest CTDI formalism should also be extended to a *nT*s of 40 mm. The *CTDI*_*w*_ calculated with the latest CTDI formalism was found to differ by −20% compared to the previous CTDI formalism, for an *nT* of 160 mm (used in, e.g., whole‐organ perfusion); however, it is important to consider that the radiation exposure to the patient and the image quality of the examination will remain the same, using the latest CTDI formalism. The real‐time solid‐state detector method provided results that differed by as much as 8% ($CTDI_{free\;air})\;$compared to the liquid ionization chamber method. The pencil ionization chamber was considered to be the most clinically feasible method that was tested and provided results that closely matched, within 2%, the liquid ionization chamber method (dosimetric reference).

## CONFLICT OF INTEREST

Love Kull is a majority shareholder in LoniTech AB. Mats Danielsson has a research collaboration with GE Healthcare and is a shareholder in Prismatic Sensors AB. The remaining authors have no conflicts of interest.

## ACKNOWLEDGMENTS

We thank the Department of General Radiology at the Karolinska University Hospital for allowing us to acquire measurements on their scanner. We thank the Swedish National Metrology Laboratory for assistance in calibrating the pencil ionization chamber and the liquid ionization chamber as well as GE Healthcare and RTI Electronics for helpful discussions about their equipment.
